# Brain Multimodality Monitoring: A New Tool in Neurocritical Care of Comatose Patients

**DOI:** 10.1155/2017/6097265

**Published:** 2017-05-07

**Authors:** Nudrat Tasneem, Edgar A. Samaniego, Connie Pieper, Enrique C. Leira, Harold P. Adams, David Hasan, Santiago Ortega-Gutierrez

**Affiliations:** ^1^Department of Neurology, Stroke Division, University of Iowa Carver College of Medicine, Iowa City, IA, USA; ^2^Department of Neurosurgery, University of Iowa Carver College of Medicine, Iowa City, IA, USA

## Abstract

Neurocritical care patients are at risk of developing secondary brain injury from inflammation, ischemia, and edema that follows the primary insult. Recognizing clinical deterioration due to secondary injury is frequently challenging in comatose patients. Multimodality monitoring (MMM) encompasses various tools to monitor cerebral metabolism, perfusion, and oxygenation aimed at detecting these changes to help modify therapies before irreversible injury sets in. These tools include intracranial pressure (ICP) monitors, transcranial Doppler (TCD), Hemedex™ (thermal diffusion probe used to measure regional cerebral blood flow), microdialysis catheter (used to measure cerebral metabolism), Licox™ (probe used to measure regional brain tissue oxygen tension), and continuous electroencephalography. Although further research is needed to demonstrate their impact on improving clinical outcomes, their contribution to illuminate the black box of the brain in comatose patients is indisputable. In this review, we further elaborate on commonly used MMM parameters, tools used to measure them, and the indications for monitoring per current consensus guidelines.

## 1. Introduction

Clinical presentation of acute brain injury (ABI) frequently includes a variable degree of altered mental status in conjunction with a very limited neurological exam. Unfortunately, these patients are at risk for further deterioration due to inflammation, edema, and ischemia triggered by primary insult. This downstream injury is called secondary brain injury (SBI) and it is often missed in unresponsive and sedated neurocritical patients. Cutting-edge technology now provides sophisticated tools that allow us to gather real-time integrated information of the pathophysiological processes in comatose patients, known as multimodality monitoring (MMM). The goal of MMM is early detection of SBI by monitoring changes in physiologic parameters that reflect cell death and injury. These parameters include intracranial pressure (ICP), cerebral perfusion pressure (CPP), cerebral blood flow (CBF), brain tissue oxygenation, cerebral metabolism, and electrocortical activity (see Table 1). The information obtained from these tools, when integrated in clinical decision making and early goal-directed therapy, might help to prevent SBI before irreversible injury occurs.

In this review, we further elaborate on commonly used MMM parameters, tools used to measure them, and indications for monitoring per current consensus guidelines [1].

## 2. Intracranial Pressure (ICP) and Cerebral Perfusion Pressure (CPP)

**Figure 1:**
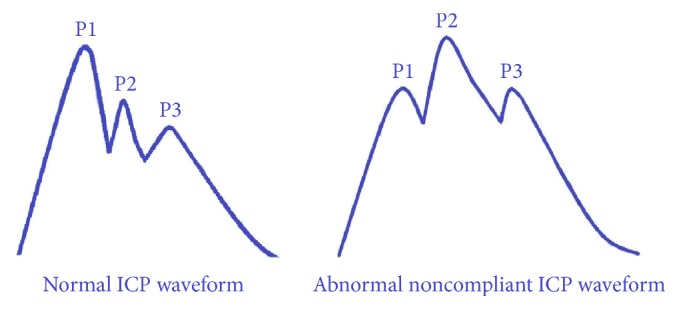
Intracranial pressure (ICP) waveforms. Percussion wave (P1) represents arterial pulsation, tidal wave (P2) represents brain tissue compliance, and dicrotic wave (P3) is due to closure of aortic valve. Under normal conditions, P1 > P2, indicative of normal compliant brain. In ABI brain compliance starts decreasing resulting in reversal of P1 : P2 ratio (i.e., P2 > P1) which is a sensitive predictor of poor brain compliance.

ICP and CPP are the most commonly monitored parameters in patients with acute brain injury. Brain parenchyma, cerebral blood volume, and cerebrospinal fluid represent normal intracranial constituents, which are contained in a nonelastic bony structure. The modified Monro-Kellie doctrine states that sum of intracranial volumes is constant and an increase in one is offset by decrease in one or both of the remaining two [1]. This principle acts as a buffer for small increases in volume with minimal change in ICP. However, in acute brain injury (either traumatic or vascular) large increase in volumes in the form of cerebral edema or expanding hematoma sets the equilibrium at a higher ICP which could produce reduction in cerebral blood flow and eventually ischemia and cerebral herniation. Normal range of ICP in adults lies between 7 and 15 mmHg. ICP values over 20–25 mmHg are indicative of intracranial hypertension [2]. Besides the absolute number, ICP waveform should also be assessed as it gives important information about proper placement of the probe and brain compliance status (Figure 1). There is enough evidence to support that sustained ICP > 20 mmHg and particularly refractory to treatment is associated with worse outcome [3, 4].

CPP is the difference between mean arterial pressure and ICP. It represents the pressure gradient driving cerebral blood flow (CBF) and hence oxygen and metabolite delivery. It is also believed to be the metric to which brain's autoregulatory mechanisms respond [5]. Normal adult CPP > 50 mmHg. Per recent brain trauma foundation guidelines, recommended CPP for survival and favorable outcome is between 60 and 70 mmHg, with patient's autoregulatory status being the most important determinant of minimal CPP threshold. Level III recommendation has also been made to avoid aggressive use of fluids and pressors to keep CPP above 70 due to risk of adult respiratory distress syndrome [6]. However, management based upon target CPP rather than ICP has not shown better outcome [7, 8]. In fact, it has been postulated that CPP values should be individualized based upon the disease state and information gathered by ICP, oxygenation, and metabolic monitoring. A recent retrospective cohort study analyzed trends in adherence to current guidelines in TBI patients and 2-week mortality. They found a significant decrease in two-week postinjury mortality with increased adherence to guidelines particularly in those where management was guided by both ICP and CPP monitoring [9].

Brain trauma foundation (3rd edition) and MMM consensus guidelines [2, 10] recommend ICP and CPP monitoring in all patients with ABI who have a Glasgow coma scale of 8 or below and/or who are at risk of elevated ICP based upon clinical and/or imaging features. However, these recommendations were not carried forward in 4th edition of brain trauma foundation guidelines, as these were derived from either descriptive studies or studies which did not meet their inclusion criteria. Current guidelines recommend management of severe TBI patients using information from ICP monitoring to reduce in-hospital and 2-week postinjury mortality (level IIB) [6].

Noninvasive tools for assessment of ICP include transcranial Doppler with pulsatility index, pupillometry, and ultrasound measurement of optic nerve sheath diameter. However, these are not commonly used in clinical practice due to their limited accuracy and interpretation compared to invasive monitoring [11]. Currently recommended devices include intraventricular catheter also known as external ventricular drainage (EVD) or intraparenchymal monitors [2]. EVD gives the most accurate assessment of global ICP; it can be recalibrated to minimize measurement drift [12], is cost effective, and allows therapeutic intervention in poorly compliant brains by drainage of CSF in cases of hydrocephalus. Disadvantages include difficult insertion especially in compressed or displaced ventricles, obstruction of fluid column, for example, by blood clot, leading to inaccurate measurements, and need to maintain the transducer at a fixed reference point relative to patient's head. Significant clinical bleeding after EVD placement and EVD related infections occur in less than 1% and 5–15%, respectively [13].

Intraparenchymal pressure sensors are easier to place and provide continuous monitoring compared to EVD, where drain system must be closed to measure ICP. The prevailing current technology includes piezoelectric strain gauge (Codman™ microsensor and Raumedic™ Neurovent) and fiberoptic (Integra Camino™) sensors. These devices need to be inserted 1.5 to 2 cm into the brain parenchyma through a burr hole. Optimal positioning close to the area at risk is of paramount importance particularly in focal lesions, since interhemispheric variations of over 10 mmHg have been described in focal lesions with mass effect. Hence CT imaging after positioning is usually recommended [14]. Of note Codman™ and Neurovent™ are MRI compatible. Intraparenchymal monitors are more expensive; measurements drift with time and cannot be recalibrated.

Other less accurate monitors include subarachnoid screw and epidural fiberoptic catheters, which are rarely used in clinical practice.

**Figure 2:**
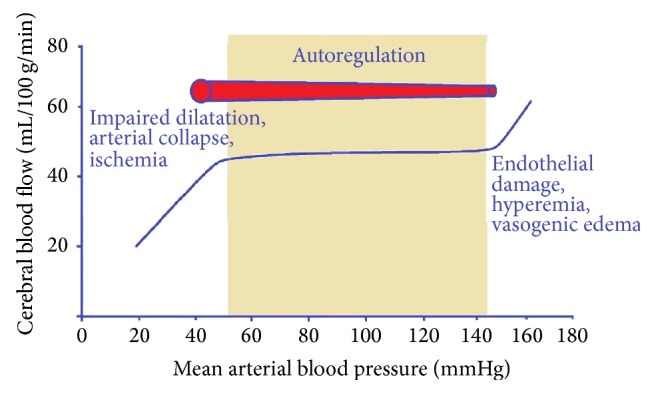
(Not to scale) Cerebral Autoregulation Curve. Autoregulation ensures nearly constant CBF despite changes in perfusion pressure over a certain range (~50–150 mmHg). In healthy brain over 150 mmHg there is endothelial damage, leading to impaired vessel reactivity resulting in hyperemia, vasogenic edema, and intracranial hypertension. Under 50 mmHg CBF becomes directly proportional to perfusion pressure with risk of arterial collapse and ischemia.

Autoregulation is another important aspect of cerebral perfusion monitoring. An uninjured brain is capable of maintaining fairly constant cerebral blood flow despite fluctuations in perfusion pressures by varying intracerebral vessel caliber (Figure 2). In an injured brain this autoregulatory mechanism is deranged putting the patient at risk for SBI via ischemia with hypotension and conversely to elevated ICP and hyperemia with MAP augmentation. This adaptive characteristic of brain vasculature can be quantified as static autoregulation, a concept reflected by the index of pressure reactivity (PRx). PRx measures the correlation between arterial blood pressure and intracranial pressure waves and reflects cerebral autoregulation in response to blood pressure changes. The PRx is scaled as a correlation coefficient (from +1.0 to −1.0), with positive values indicating linear correlation with changes in MAP, reflecting an impaired autoregulatory state. A retrospective cohort study of 398 patients showed lower mortality with PRx value of <0.25 (20% versus 69%) [15].

## 3. Cerebral Blood Flow (CBF)

Neuroimaging modalities particularly perfusion CT or MR are frequently used in clinical practice for estimating cerebral blood flow [11]. However, they provide a snap shot in time, whereas CBF is a dynamic process. Thus, supplementing neuroimaging with continuous monitoring at bedside may provide a more comprehensive picture of cerebral perfusion status.

CBF can be monitored noninvasively using transcranial Doppler (TCD) which gives more global assessment by measuring the mean flow velocities in different intracerebral vessels. TCD is primarily used to detect vasospasm in SAH and hence identify patients at risk for delayed ischemia. It is more reliable for evaluating anterior circulation and a mean MCA flow velocity of >200 cm/s has a high probability of predicting clinically significant vasospasm [14]. However increased velocity can reflect vasospasm (i.e., decreased diameter) or hyperemia. Lindegaard ratio (LR), which is the ratio of highest flow velocity in MCA to highest flow velocity in external ICA, helps differentiate between hyperperfusion and vasospasm and LR value > 3 is considered accurate to differentiate between the two [16]. Predictive power of TCD especially for vessels difficult to insonate (ICA and ACA) can be improved with transcranial color coded duplex sonography [17]. Limitations of TCD include operator based variability and inability to differentiate symptomatic versus asymptomatic vasospasm, especially at velocity between 120 and 199 cm/s [14].

CBF can also be measured by inserting a thermal diffusion probe (TDP) directly into brain parenchyma. The commercially available system includes the Hemedex™ monitoring system, which is not MRI compatible. It permits regional CBF (rCBF) monitoring by assessing thermal convection due to tissue blood flow. The probe tip is inserted into white matter of brain and its utility depends on proximity to the area of interest. TDP has been validated by Xenon perfusion CT [18] and CBF level below 15 mL/100 g/min is identified as threshold for diagnosis of hypoperfusion [19]. Per MMM consensus guidelines TDP should be placed in vascular territory of ruptured aneurysm to monitor for vasospasm [2]. Quantification of rCBF with TDP is highly dependent on patient's core body temperature and is significantly altered in conditions of hyperthermia.

To date, there are no published studies of improved outcome with treatment strategies directed solely by CBF monitoring but it seems to be a promising tool to use in conjunction with other parameters.

## 4. Cerebral Oxygenation

Maintenance of adequate oxygenation is vital for critically ill neurologic patients. Brain oxygenation is a surrogate of CBF and in conjunction with metabolic parameters serves as a marker of tissue at risk for ischemia. Brain tissue oxygen tension (PbtO_2_) is the product of CBF and cerebral arteriovenous oxygen tension difference [20]. PbtO_2_ is used as adjunct with ICP monitoring in guiding management of CPP and tailoring individual CPP threshold in patients with ABI [20]. PbtO_2_ is an invasive means of monitoring regional cerebral oxygen tension by inserting a microcatheter in the white matter, in the region at high risk for ischemia as determined by CT or MRI perfusion studies. There are two commercially available probes for monitoring PbtO_2_, Licox™ system (which provides additional ICP and brain temperature monitoring) and the Neurovent-PTO™ system (which measures partial pressure of carbon dioxide and PH as well). Both measure oxygen content in adjacent white matter and are safe and efficacious but cannot be used interchangeably as significant difference in measured PbtO_2_ values was observed when comparing the two devices [21]. Normal PbtO_2_ is 23–35 mmHg with Licox™ [22]. Current MMM guidelines consider PbtO_2_ of less than 20 mmHg as threshold to consider intervention [2].

SjVO_2_ monitoring requires retrograde insertion of special fiber optic catheter in the origin of internal jugular vein at the skull base, preferably in dominant vein to assess global oxygenation. SjVO_2_ reflects the difference between cerebral oxygen supply and demand, given that arterial hemoglobin saturation and concentration remain stable [23]. Normal levels are 60–75%. Desaturation to less than 50% suggests ischemia. Multiple or sustained (>10 minutes) desaturations are associated with poor outcome in TBI patients [24]. SjVO_2_ above 75% indicates hyperemia or infarcted tissue. Sampling of blood from the catheter gives jugular vein oxygen content, which with arterial blood oxygen content is used to calculate arterial-jugular venous oxygen content difference (AVDO_2_). AVDO_2_ above 9 mL/dL probably indicates global cerebral ischemia and values less than 4 mL/dL indicate hyperemia. Use of SjVO_2_ is limited by need for frequent recalibrations and catheter related complications including infection, elevated ICP, thrombosis of the vein, and pneumothorax [23]. Secondly small areas of regional ischemia may not produce any change in SjVO_2_, as it is a reflection of global cerebral oxygenation.

SjVO_2_ or PbtO_2_ monitoring is indicated in patients requiring hyperventilation to control ICP (PCO_2_ 20–25) [12] and is also instrumental in patients at risk of cerebral ischemia or hypoxia [2]. PbtO_2_ can also be used as an adjunct with TCD to monitor for delayed cerebral ischemia in comatose SAH patients [20]. In TBI patients, which is thought to be a diffuse process, it is recommended to place the probe at the least injured site. In SAH probe should preferentially be placed in region at highest risk for vasospasm (which is the vascular territory of ruptured aneurysm) and in intracerebral hemorrhage probe should be placed near the site of hemorrhage. PbtO_2_ monitoring and directed therapy has been shown to improve long term functional outcome in poor grade aneurysmal SAH [25]. Current MMM guidelines suggest SjVO_2_ or PbtO_2_ monitoring to assist ICP/CPP directed therapy, identify refractory intracranial hypertension and treatment thresholds, help manage delayed cerebral ischemia, and select patients for second tier therapy for persistent intracranial hypertension [2].

Recent brain trauma foundation guidelines (4th edition) recommend jugular bulb monitoring for arteriovenous oxygen content difference to help guide management decisions (level III). Moreover, brain tissue oxygenation <15 mmHg as treatment threshold from was removed current recommendations, as available evidence was not sufficient for formal recommendation and a recent retrospective cohort of 629 patients showed no difference in mortality rate for TBI patients who were managed with ICP and PbtO_2_ monitoring versus ICP monitoring alone [6, 26].

Near infrared spectroscopy (NIRS) is an emerging noninvasive tool to measure cerebral oxygenation. It calculates the concentration of a chromophore (oxygenated hemoglobin in brain injury patients, rSO_2_) based upon attenuation of light between the light source and receiver. A study on 94 randomly selected healthy adults reported mean cerebral oxygen saturation of 67.14 ± 8.84% using NIRS [27]. However, to date no studies have been done to establish rSO_2_ thresholds predictive of SBI. Another important limitation of NIRS is the contamination of signal by scalp swelling and epidural/subdural hematomas (which are common in TBI patients) leading to unreliable measurements.

## 5. Cerebral Metabolism

Although brain tissue oxygen, CBF, and CPP monitoring provide critical physiological information, monitoring of various substrates, metabolites, and neurotransmitters during the course of acute brain injury can provide additional insight into the pathophysiological processes and ultimate mitochondrial derangement that impair oxidative metabolism. This information when combined with data gathered from ICP, CBF, and PbtO_2_ monitoring can help guide therapy to minimize further brain injury.

Neuroimaging particularly PET scan and MR spectroscopy provide information regarding glucose uptake and lactate content, respectively [23]. However, these imaging modalities provide static information whereas cerebral metabolism is a dynamic process. Secondly, most of the patients are critically ill and cannot be transported back and forth to obtain these images. The advent of cerebral microdialysis (CMD) has revolutionized the monitoring of cerebral metabolism. With microdialysis various substrates, neurotransmitters, and metabolites can be analyzed at hourly intervals at the bedside. Current MMM guidelines recommend cerebral microdialysis in patients with or at risk for ischemia, hypoxia, and energy failure [2]. They also suggest using CMD to assist titration of medical therapies like systemic glucose control, transfusion, and therapeutic hypothermia [2]. A single center prospective study of 165 patients addressed the use of information obtained from CMD monitoring to manage TBI patients and found reduced mortality and better outcome at 6 months in patients whose glutamate normalized within 120 hours of monitoring [28]. But recent brain trauma foundation guidelines have not found sufficient evidence to support any level of recommendation [6].

The microdialysis catheter is 0.62 mm wide, lined with semipermeable membrane with a pore size of typically 20 kDa. The catheter is inserted into subcortical white matter and perfused with either normal saline or ringer's solution at very slow rates (0.1–2.0 microliters per minute) with a pump system. Molecules below the membrane cut-off size diffuse down their concentration gradient and equilibrate with the perfusion fluid. This fluid is collected in vials and analyzed hourly by either enzyme spectrophotometry or high performance liquid chromatography.

The clinical application of microdialysis in neurocritical care is primarily focused on delivery and metabolism of glucose. Under normal conditions, that is, aerobic conditions, glucose gets metabolized to pyruvate and adenosine triphosphate. A decrease in glucose could be due to reduced perfusion, decreased systemic supply, or increased utilization. Elevated glucose, on the other hand, could be due to hyperemia, increased systemic levels, or decreased metabolism. Under hypoxic conditions or impaired mitochondrial functioning (which is common in ischemic injury), glucose gets metabolized to lactate. In fact, lactate, pyruvate, and lactate to pyruvate ratio are considered markers of anaerobic metabolism and energy crisis, with LPR being more reliable of all three [29]. Glutamate, an excitatory neurotransmitter, is associated with ischemia, inflammation, and cell damage. It is one of the earliest markers of vasospasm compared to other substrates [30]. Glycerol is an integral part of neuronal structure and elevated level signifies ischemia that has progressed to cell damage [29].

Average concentration of glucose, lactate, and pyruvate in normal adults under sedation is reported as 1.7 ± 0.9 mmol/L, 2.9 ± 0.9 mmol/L, and 166 ± 47 *μ*mol/L [31] and LPR of >40 has been reported as marker of metabolic distress in TBI [31]. However, in patients with acute brain injury, it is the trend rather than absolute value of these substrates, which in conjunction with other parameters helps guide therapeutic strategies.

Microdialysis catheter should be placed perilesionally in focal brain injuries, in right frontal region for diffuse TBI, and in ACA-MCA watershed region on the side of aneurysm rupture for SAH [31]. Poor outcomes have been associated in patients with severe TBI with metabolic derangements seen by CMD with particular evidence for low glucose and LPR [29]. In SAH patients with delayed cerebral ischemia, lactate and glutamate rise early followed by glycerol. Elevated LPR has been reported to precede clinically delayed cerebral ischemia by 11 to 13 hours in patients with SAH [29].

## 6. Electroencephalography (EEG)

EEG provides information about brain electrical activity and is indicated to detect seizures. Most commonly, electrodes are applied to the scalp which record activity of cerebral cortex. However, in certain instances electrodes can also be applied directly on the brain surface, which is more sensitive than scalp EEG to detect seizures.

Neurocritical care patients often have nonconvulsive seizures, which are subclinical. The prevalence of nonconvulsive seizures in patients with brain injury including TBI, SAH, ICH, and hypoxic-ischemic encephalopathy ranges from 4 to 30% [32] and is associated with secondary cerebral damage, evidenced by elevated LPR and ICP [33]. Continuous EEG for a minimum of 48 hours is required to detect nonconvulsive seizures with >90% sensitivity among comatose patients. Nonconvulsive seizures are associated with increased morbidity and mortality regardless of etiology [34].

Current MMM guidelines recommend EEG in all patients with ABI and unexplained altered consciousness, in patients with convulsive status epilepticus who do not return to baseline within 60 minutes after medication, during therapeutic hypothermia and within 24 hours after rewarming [2].

Besides seizures, certain EEG patterns like broad repetitive slow waves were found to highly correlate with occurrence of vasospasm in SAH, which lead to the development of quantitative EEG (qEEG). qEEG is data obtained from processing hours long of raw EEG data using compressed spectral array. The variable recorded like alpha/delta ratio, power, and alpha variability can be utilized to detect delayed cerebral ischemia in SAH [2, 11, 35].

Despite its widespread use, conventional scalp EEG has its limitations especially in ICU setting. Poor signal-to-noise ratio, poor spatial resolution, suboptimal electrode to scalp contact, and interference from electrical devices are all factors that hamper the interpretation of scalp EEG. Sometimes patterns are suspicious but not diagnostic of ictal events. Given these limitations, the concept of intracortical depth electrodes has been introduced. Small scale studies have shown that intracortical depth electrodes can detect seizures and cortical spreading depression that cannot be seen on scalp EEG [11, 36]. The placement of depth electrode has safety profile similar to other invasive monitoring devices and can potentially be used to identify early changes in brain activity indicative of SBI [36]. It involves insertion of 6 or 8 contact depth electrodes either through a dedicated burr hole or with the EVD, with contacts distributed over both gray and white matter. The rate of seizure detection is much higher than that detected by surface EEG, and baseline muscle artifact is completely eliminated with this technique. However larger studies are required to evaluate full potential of depth electrodes and assess the outcome of therapy based upon such monitoring.

## 7. Integration of the MMM Information and Conclusion

**Figure 3:**
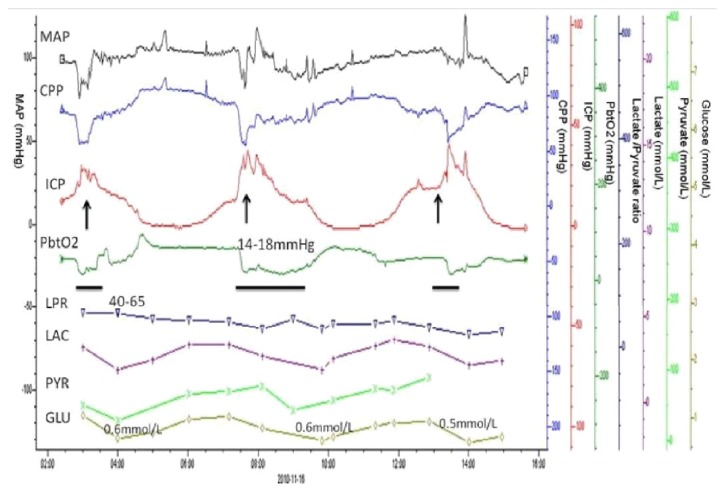
Real-time relationship of patient's physiological parameters with acute brain injury. (A) As ICP plateau waves occur (arrows), simultaneous drops in CPP below 60 mmHg and PbtO_2_ below 15 mmHg were observed. Microdialysis data consistently showed elevated LRP but consistent decrease in brain glucose levels occurred after each plateau wave, suggesting metabolic disturbance after brain hypoxia secondary to cerebral flow failure.

With the advancement of technology and informatics, data collection no longer represents a problem. The aim of multimodality neuromonitoring is not to add new variables for an intensivist to chase but to integrate information from multiple modalities to formulate a patient-specific “injury profile” which will guide formulation of an optimal treatment plan.  Figure 3 shows an example of how information from various modalities can be integrated to determine optimum patient-specific thresholds. The figure depicts real-time monitoring of a patient's physiologic parameters and the effect of elevations in ICP on CPP and PbtO_2_ resulting in regional hypoxia reflected by decrease in brain glucose levels.

Given the complexity of the data and the need for global interpretation of these parameters, acquisition systems, which allow complete integration of all the parameters including MMM, vital signs, cEEG, and temperature, are of paramount importance. Currently, there is only one commercially available system called CNS monitor (Moberg Research) that only allows monitoring of a single patient at any given time. Further advances in integrated systems with optimal signal-to-noise ratios that allow perfected event detection algorithms are necessary to move to a complete integrated approach.

Nevertheless, MMM is now a reality commonly used in advance neurocritical care units throughout the world. Although various studies have shown the physiologic feasibility of monitoring various neurologic parameters, there is still no published data from randomized trials to support that targeting any variable improves clinical outcome. Steiner et al. [28] analyzed the relationship of MAP and ICP to identify an optimum CPP in 114 TBI patients and demonstrated that patients with a mean CPP closer to their “optimum” CPP target were more likely to have a favorable outcome. Soehle et al. [37] studied the relationship between CPP and PbtO_2_ to quantify impairment of cerebral autoregulation. Nevertheless, further larger randomized trials are needed to demonstrate their impact on clinical outcome.

Ideally research using multimodality bedside monitoring will help identify patient-specific physiologic thresholds, which will enable neurointensivists to optimize patient's physiology and minimize further secondary neurologic injury. As bioinformatics continues to advance, further improvement in systems providing physicians with real, accurate information of individual physiological states is only a question of time.

## Figures and Tables

**Table 1:** Multimodality parameters: commonly used measurement devices, physiologic ranges, threshold at which early goal therapy should be considered, and clinical significance.

Modality	Means of monitoring	Physiologic range	Threshold	Clinical significance
Intracranial pressure	(1) Intraparenchymal monitor	<20 mmHg	>20–25 mmHg	Marker of cerebral edema and impending herniation.
	(2) Intraventricular monitor (EVD)			

Cerebral perfusion pressure		60–70 mmHg	<60 mmHg	Indirect surrogate of CBF. Guide treatment of intracranial hypertension to optimize perfusion.

Cerebral blood flow	(1) TCD	Mean flow velocities	MCA mean flow velocity >200 cm/s	Detection of vasospasm and delayed cerebral ischemia in SAH.
		MCA 30–75 cm/s		
		ACA 20–75 cm/s		
		PCA 15–55 cm/s		
		LR < 3	LR > 6	Differentiate hyperemia from vasospasm.
	(2) TDP	50 mL/100 g/min	<20 mL/100 g/min	Indicative of regional cerebral ischemia.

Cerebral oxygenation	(1) Juglar venous oximetry	50–80%	<50% or >80%	Indicative of global ischemia or hyperemia and tissue extraction of oxygen.
	(2) Licox™	35–40 mmHg	<20 mmHg	Indicative of regional hypoxia/hypoperfusion.

Cerebral metabolism	Microdialysis	Glucose 0.4–4.0 *μ*mol/L	<0.4	Indicative of brain energy supply and demand.
		Lactate 0.7–3.0 *μ*mol/L	>3.0	
		Pyruvate unknown Lactate to pyruvate ratio <20	>40	Elevated LPR indicative of ischemia, anaerobic metabolism.
		Glutamate 2–10 *μ*mol/L	>10	Increased glutamate and lactate earliest marker of ischemia followed by increased glycerol.
		Glycerol 10–90 *μ*mol/L	>90	

TCD: transcranial cranial doppler; TDP: thermal diffusion probe; MCA: middle cerebral artery; ACA: anterior cerebral artery; PCA: posterior cerebral artery; SAH: subarachnoid hemorrhage; LR: Lindegaard ratio; LPR: lactate to pyruvate ratio.
